# Production, purification, and crystallization of the recombinant HER2 tyrosine kinase domain (HER2-TKD)

**DOI:** 10.55730/1300-0527.3794

**Published:** 2026-04-10

**Authors:** Edanur TOPALAN, Halilibrahim ÇİFTÇİ, Hasan DEMİRCİ

**Affiliations:** 1 Department of Molecular Biology and Genetics, Faculty of Science, Koç University, İstanbul, Turkiye; 2 Medicinal and Biological Chemistry Science Farm Joint Research Laboratory, Faculty of Life Sciences, Kumamoto University, Kumamoto, Japan; 3 Department of Drug Discovery, Science Farm Limited, Kumamoto, Japan; 4 Department of Molecular Biology and Genetics, Faculty of Science and Letters, Burdur Mehmet Akif Ersoy University, Burdur, Turkiye; 5 Stanford Photon Ultrafast Laser Science and Engineering Institute, Stanford Linear Accelerator Center National Laboratory, Menlo Park, CA, USA

**Keywords:** HER2-TKD, soluble expression, sarcosyl, inclusion bodies, protein crystallization, *Escherichia coli*

## Abstract

The human epidermal growth factor receptor 2 tyrosine kinase domain (HER2-TKD) is an important therapeutic target in oncology. In this study, we developed a practical and cost-effective approach for producing soluble recombinant HER2-TKD in *Escherichia coli* as a basis for structure-based drug discovery. The gene encoding HER2-TKD was cloned into a pET28a(+) expression vector and expressed in *E. coli*. Since the protein was initially obtained as inclusion bodies, solubilization with 1.5% sarcosyl was applied to recover it in soluble form. The purified protein was obtained through size-exclusion chromatography, followed by tag removal using reverse affinity purification. Crystallization trials were performed under more than 3000 conditions with commercial screening kits. Although most of the early crystals turned out to be salts rather than protein crystals, these observations underline the inherent challenges of HER2-TKD crystallization and highlight areas for further optimization. Overall, we establish a reproducible expression and purification workflow for HER2-TKD and provide a foundation for future cocrystallization experiments with small-molecule inhibitors and applications in structure-based drug screening.

## 1. Introduction

The epidermal growth factor receptor 2 (HER2), also known as ErbB2, is a member of the ErbB family of receptor tyrosine kinases (RTKs), which are key regulators of cellular proliferation, differentiation, and survival. HER2 plays a critical role in cancer biology, particularly in breast cancer and non-small cell lung cancer (NSCLC) [1]. In many cancers, mutations in the tyrosine kinase domain (TKD) of HER2 are frequently detected. These mutations often induce conformational alterations in the ATP-binding pocket (ATP-BP), resulting in increased kinase activity and activation of oncogenic signaling pathways [2–6].

Unlike other ErbB family members that require ligand binding for dimerization and activation, HER2 is constitutively active in a ligand-independent manner [7]. Its potent ability to form heterodimers with other ErbB receptors enhances its signaling capacity, making HER2 deregulation a significant driver of tumorigenesis [1]. In breast cancer, HER2 amplification is often the primary oncogenic event. In contrast, HER2-driven NSCLC involves more diverse mechanisms [8–10], including gene amplification, activating mutations [11–13], and overexpression [14–17].

Under physiological conditions, HER2 activity is tightly regulated by a balance between its phosphorylated (active) and dephosphorylated (inactive) forms, which is essential for normal cellular growth and breast tissue development [18]. However, when this balance is disrupted by overactivation or mutation, it leads to uncontrolled cell proliferation and survival [19–23]. Although HER2-targeted monoclonal antibodies (mAbs) and earlier-generation pan-HER tyrosine kinase inhibitors (TKIs) have shown limited efficacy in treating HER2-altered NSCLC, newer HER2-specific TKIs have demonstrated more encouraging results [24–26]. Nonetheless, resistance mechanisms, such as the activation of alternative RTK pathways and upregulation of downstream survival signals, remain a challenge for both TKIs and mAbs [27,28]. In the present paper, we describe the successful expression of HER2-TKD in *Escherichia coli*, where the protein first forms inclusion bodies. The recombinant HER2-TKD was solubilized in 1.5% sarcosyl, purified using affinity and size-exclusion chromatography, and verified by SDS-PAGE. Crystallization investigations commenced utilizing commercially available sparse matrix screens to acquire diffraction-quality crystals. The present paper outlines a reproducible and effective technique for the creation and purification of soluble HER2-TKD, making it appropriate for structural investigations. Creating this platform is crucial for enabling structure-based drug discovery targeting HER2, especially in cancers with abnormal HER2 activity.

## 2. Materials and methods

### 2.1. Preparation for protein expression

#### 2.1.1. Gene construct design

HER2-TKD:

**MSDSEVNQEAKPEVKPEVKPETHINLKVSDGSSEIFFKIKKTTPLRRLMEAFAKRQGKEMDSLRFLYDG IRIQADQTPEDLDMEDNDIIEAHREQIGG**MSGAAPNQALLRILKETELRKVKVLGSGAFGTVYKGIWIP DGENVKIPVAIKVLRENTSPKANKEILDEAYVMAGVGSPYVSRLLGICLTSTVQLVTQLMPYGCLLDHV RENRGRLGSQDLLNWCMQIAKGMSYLEDVRLVHRDLAARNVLVKSPNHVKITDFGLARLLDIDETEYHA DGGKVPIKWMALESILRRRFTHQSDVWSYGVTVWELMTFGAKPYDGIPAREIPDLLEKGERLPQPPICT IDVYMIMVKCWMIDSECRPRFRELVSEFSRMARDPQRFVVIQNEDLGPASPLDSTFYRSLLEDDDMGDL VDAEEYLVPQQGAAAS*

**Bold:** Sumo tag

The plasmid construct containing the coding sequence of the human HER2 tyrosine kinase domain (UniProt ID: P04626) was obtained from GenScript (Piscataway, NJ, USA). The sequence was cloned into the pET28a(+) expression vector, which carries a kanamycin resistance marker. The construct included an N-terminal 6xHis tag to facilitate purification and a SUMO tag to enhance solubility. The plasmid was supplied in ready-to-use form and was subsequently used for transformation into *E. coli* Rosetta 2 (DE3) pLysS cells.

#### 2.1.2. Transformation procedure

Two micrograms of the HER2 plasmid were dissolved in 25 μL of distilled water and the solution was vortexed for 30 s. The mixture was centrifuged at 9000 rpm for 4 min, and the vortexing and centrifugation steps were repeated to ensure homogeneity. The plasmids prepared were stored at −20 °C until use.

For transformation, 2 μL of the plasmid DNA was mixed with 50 μL of *E. coli* Rosetta 2 (DE3) pLysS competent cells that had been thawed on ice [29]. The mixture was incubated on ice for 20 min, subjected to a heat shock at 42 °C for 45 s [30], and immediately placed on ice for 2 min. Then 500 μL of sterile LB medium was added and the cells were incubated at 37 °C for 90 min, with gentle inversion every 15 min. After incubation, 50 μL of the culture was spread onto LB agar plates containing kanamycin (50 μg/mL) and chloramphenicol (35 μg/mL). The plates were incubated overnight at 37 °C.

The next day, individual colonies were selected and inoculated into 10 mL of LB broth, which was cultivated overnight at 37 °C. For glycerol stock preparation, 750 μL of the overnight culture was mixed with 750 μL of sterile 80% glycerol in cryotubes. After vortexing to ensure homogeneity, the glycerol stocks were stored at −80 °C.

### 2.2. Minichecks

#### 2.2.1. Miniscale production of HER2 protein and incubation time optimization

Following transformation, protein expression was evaluated. A 10-mL volume of LB medium was sterilized by autoclaving and, once cooled, antibiotics were added at a 1:1000 ratio (10 μL of kanamycin and 10 μL of chloramphenicol). Chloramphenicol was used because *E. coli* Rosetta 2 (DE3) pLysS cells carry chloramphenicol resistance, while kanamycin was used to select for the pET28a(+) plasmid. After antibiotic addition, a portion of the HER2 glycerol stock was inoculated into the medium, followed by incubation overnight at 37 °C.

On the following day, protein expression was induced by adding 0.4 M IPTG at a 1:1000 dilution (final concentration 0.4 mM). During incubation at 37 °C, 1-mL samples were collected 1, 2, 3, 4, 5, 6, 7, 8, and 16 h postinduction. The samples were centrifuged at 6000 rpm for 5 min, and the resulting pellets were resuspended in 750 μL of lysis buffer and subjected to sonication. Finally, 40 μL of each lysate was mixed with 10 μL of loading dye, prepared for gel analysis, and analyzed by 12% SDS-PAGE using a Mini-PROTEAN Tetra Vertical Electrophoresis Cell (Bio-Rad Laboratories, Hercules, CA, USA).

#### 2.2.2. Solubility analysis of HER2 protein in miniscale production

The culture was grown in 10 mL of LB medium at 37 °C until an OD_600_ of 0.6 was achieved, at which point expression was induced with 0.4 mM IPTG and incubation continued for 7 h. The resulting 10 mL of culture was equally divided into two 5-mL aliquots and harvested by centrifugation.

To evaluate the solubility of HER2 and to assess the solubilizing effect of sodium lauroyl sarcosinate (sarcosyl) in the case of the formation of inclusion bodies, we designed an experiment comparing two distinct lysis buffers: one with sarcosyl and one without. For the sample labeled “C,” lysis was performed using a buffer containing 200 mM NaCl, 20 mM imidazole, 50 mM Tris-HCl, 5% glycerol, and 0.1% Triton X-100 (pH 8.5). For the sample labeled “S,” the lysis buffer was composed of 10 mM Tris, 150 mM NaCl, 1 mM PMSF, 5 mM BME (2-mercaptoethanol), and 1.5% sarcosyl [13] (pH 8.5).

After the addition of a lysis buffer, the samples were sonicated at 41% amplitude in three cycles of 15 s each. Following sonication, whole cell lysates (WCLs) were collected for gel analysis. The samples were then centrifuged at 6000 rpm for 5 min and both pellet and supernatant fractions were prepared for SDS-PAGE. All samples were analyzed on 12% SDS-PAGE gels.

### 2.3. Large-scale protein production

Following the optimization of incubation time and solubility conditions for HER2 protein, large-scale production was performed. Four 1-L LB cultures were grown at 37 °C until an OD_600_ of 0.6 was achieved. Protein expression was induced with 0.4 mM IPTG, and incubation was continued for 7 h. At the end of the incubation period, the cultures were harvested by centrifugation at 3500 rpm for 45 min.

The resulting cell pellets were resuspended in lysis buffer (10 mM Tris, 150 mM NaCl, 1 mM PMSF, 5 mM BME, 1.5% sarcosyl) and were subjected to sonication at 41% amplitude, using 11 cycles of 20 s each.

Following sonication, the lysate was clarified by ultracentrifugation at 35,000 rpm for 1 h. To remove residual sarcosyl detergent, the supernatant was dialyzed against 2 × 1 L of dialysis buffer at 4 °C overnight. The clarified and dialyzed sample was subsequently subjected to affinity purification as described in subsection 2.3.1.

#### 2.3.1. Purification and tag removal

HER2 protein was subjected to size-exclusion chromatography (SEC) [31] using a Superdex 200 column (Cytiva, Marlborough, MA, USA) preequilibrated with 20 mM Tris-HCl (pH 8.0) and 150 mM NaCl. Elution was monitored at 280 nm and fractions were collected at 5-mL intervals. Fractions collected were analyzed by SDS-PAGE.

Fractions corresponding to the target protein were pooled and concentrated using Amicon Ultra centrifugal filters (30-kDa cutoff). The concentrated sample was incubated with ULP1 protease (5:1000, w/w) at 4 °C for 40 min to remove the N-terminal His_6_-SUMO tag [32]. After digestion, the sample was passed through a Ni-NTA affinity column (HisTrap HP, GE Healthcare) preequilibrated with binding buffer (20 mM Tris-HCl pH 8.0, 150 mM NaCl, 20 mM imidazole, 5% glycerol). The flow-through containing untagged HER2-TKD was collected. Bound proteins were removed by washing with wash buffer (20 mM Tris-HCl pH 8.0, 150 mM NaCl, 50 mM imidazole, 5% glycerol) and eluted with elution buffer (20 mM Tris-HCl pH 8.0, 150 mM NaCl, 250 mM imidazole, 5% glycerol). All fractions were analyzed by SDS-PAGE.

### 2.4. Crystallization

Crystallization trials were performed using the sitting-drop microbatch [33] under oil method in 72-well Terasaki plates (Greiner Bio-One, Kremsmünster, Austria), as previously described [29]. Purified HER2-TKD protein samples were prepared in two forms: uncleaved SUMO-tagged HER2-TKD and SUMO tag-cleaved HER2-TKD. For standard trials, 0.83 μL of protein solution was mixed with 0.83 μL of reservoir solution (1:1 ratio; total drop volume 1.66 μL), and each droplet was immediately overlaid with 16.6 μL of 100% paraffin oil (Tekkim Kimya, İstanbul, Türkiye) to prevent evaporation. Approximately 3000 unique conditions were screened at each incubation temperature using commercial sparse-matrix screens, including Natrix I–II, Wizard I–IV, Crystal Screen I–II, JCSG+, Pact Premier, Morpheus, PEG/Ion, SaltRx, ProPlex, and MembFac (Table), resulting in a total of approximately 3000 tested conditions across 4 °C and room temperature (approximately 20–22 °C). Protein samples were concentrated up to approximately 2 mg/mL and this value was therefore used as the working concentration for all trials. In optimization assays, the reservoir volume was fixed at 0.83 μL, while the protein volume was varied between 0.70 μL and 1.25 μL, generating protein-to-reservoir ratios between approximately 0.8:1 and 1.5:1. Plates were monitored daily under a polarized light microscope to evaluate crystal formation.

### 2.5. Chemicals and equipment

All chemicals, equipment, and crystallization screens used in the study were obtained from commercial suppliers. Antibiotics (ampicillin, chloramphenicol, and kanamycin), IPTG, and Ni-NTA resin were purchased from GoldBio (St. Louis, MO, USA) and Qiagen (Hilden, Germany). Other chemicals, including glycerol, NaCl, HCl, urea, and yeast extract, were obtained from ISOLAB (Eschau, Germany) and BD Biosciences (Franklin Lakes, NJ, USA). Imidazole was supplied by BioFroxx (Einhausen, Germany), while sarcosyl, SDS, Tris base, and Triton X-100 were purchased from Sigma-Aldrich (St. Louis, MO, USA). Paraffin oil was obtained from Tekkim Kimya (Bursa, Türkiye). LB agar and tryptone were purchased from Caisson Laboratories (Smithfield, UT, USA) and BD Biosciences, respectively.

The instruments used in the study included ÄKTA Go and ÄKTA systems (Cytiva, Marlborough, MA, USA), Beckman Coulter centrifuges (Allegra 15R, Avanti J-26S, and Optima L-80 XP ultracentrifuge; Beckman Coulter, Brea, CA, USA), and a Branson W250 sonifier (Branson Ultrasonics, Danbury, CT, USA). Cryotubes were obtained from Wuxi NEST Biotechnology (Wuxi, China), Eppendorf tubes and shakers (Innova 44R and 4430R) from Eppendorf (Hamburg, Germany) and New Brunswick Scientific (Edison, NJ, USA), and Falcon tubes from FıratMed (İstanbul, Türkiye). Kimtech Science KimWipes were purchased from Kimberly-Clark (Irving, TX, USA), NanoDrop 2000c from Thermo Fisher Scientific (Waltham, MA, USA), and Terasaki plates from Greiner Bio-One (Kremsmünster, Austria). For protein analysis, the Mini-PROTEAN Tetra Vertical Electrophoresis Cell (Bio-Rad Laboratories) was employed.

Crystallization trials were carried out using a variety of commercial screens, including Natrix, Wizard, Crystal Screen, JCSG+, PACT Premier, Morpheus, PEG/Ion, SaltRx, ProPlex, and MembFac screens, mainly obtained from Hampton Research (Aliso Viejo, CA, USA), Molecular Dimensions (Sheffield, UK), Rigaku Reagents (The Woodlands, TX, USA), and NeXtal Biotechnologies. X-ray diffraction data collection was performed using a Rigaku XtaLAB Synergy Flow XRD system (Rigaku Corporation, Tokyo, Japan), and purified proteins were further analyzed using a Superdex 200 Increase column (Cytiva, Marlborough, MA, USA).

## 3. Results

### 3.1. Overexpression and purification of soluble HER2-TKD

**Figure 1:**
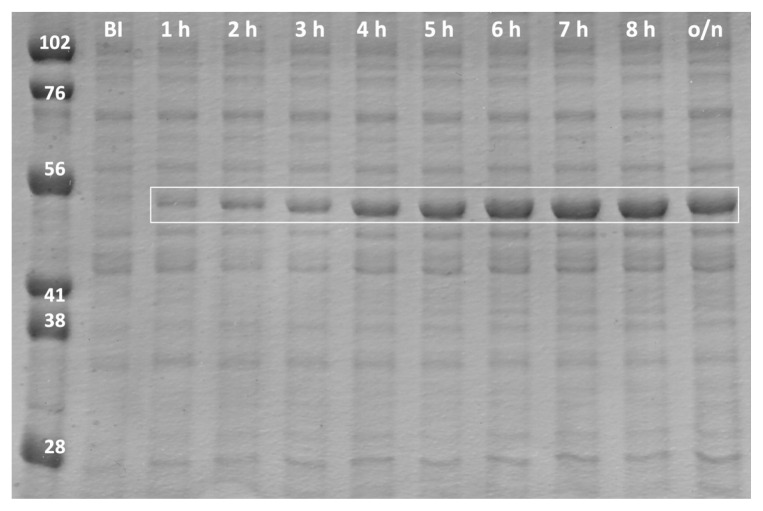
SDS-PAGE analysis of HER2 protein expression at different induction times. HER2 protein expression was evaluated after IPTG induction at various time points (1, 2, 3, 4, 5, 6, 7, 8, and overnight (o/n: 16 h). Whole-cell lysates (WCLs) corresponding to each induction time were loaded onto 12% SDS-PAGE gel. Lane 1: protein marker; lane 2: cell lysate before induction (BI). Lanes 3 to 11 show samples collected 1 h (lane 3), 2 h (lane 4), 3 h (lane 5), 4 h (lane 6), 5 h (lane 7), 6 h (lane 8), 7 h (lane 9), 8 h (lane 10), and 16 h (lane 11) postinduction. Lane 11 represents the overnight induction sample. The prominent band corresponding to HER2-TKD (approximately 48 kDa) became more intense up to 7 h of induction, after which no significant increase was observed. Based on these results, 7 h of IPTG induction was determined to be optimal for efficient HER2-TKD production. A volume of 3 μL was loaded for all samples.

**Figure 2:**
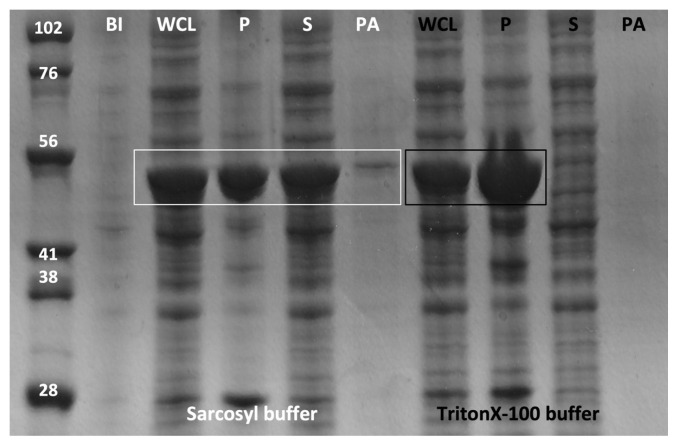
Solubility analysis of HER2 protein with and without sarcosyl detergent by 12% SDS-PAGE. HER2 protein was expressed in *E. coli* and induced with 0.4 mM IPTG at 37 °C for 7 h. After harvesting, cells were lysed using buffer systems containing either 1.5% sarcosyl (pH 8.5) or Triton X-100 (no sarcosyl), to evaluate the detergent’s effect on protein solubility. Whole-cell lysates (WCLs), soluble fractions (supernatants: S), and insoluble fractions (pellets: P) were separated by centrifugation and analyzed by 12% SDS-PAGE. Lane 1 contains the protein marker. Lanes 3–7 correspond to samples prepared in buffer with sarcosyl: before induction (lane 2), WCL (lane 3), pellet (lane 4), supernatant (lane 5), and pull-down (PA) fraction (lane 6). Lanes 8–11 show samples prepared with Triton X-100: WCLs (lane 7), pellet (lane 8), supernatant (lane 9), and pull-down fraction (lane 10). The HER2-TKD band (approximately 48 kDa) was predominantly observed in the pellet fraction with Triton X-100, indicating poor solubility. In contrast, a significant portion of HER2-TKD shifted to the soluble fraction when sarcosyl was included, demonstrating the detergent’s positive effect on solubilization. Based on these results, further HER2 protein purification was continued using sarcosyl-containing buffers. A volume of 3 μL was loaded for all samples.

**Figure 3:**
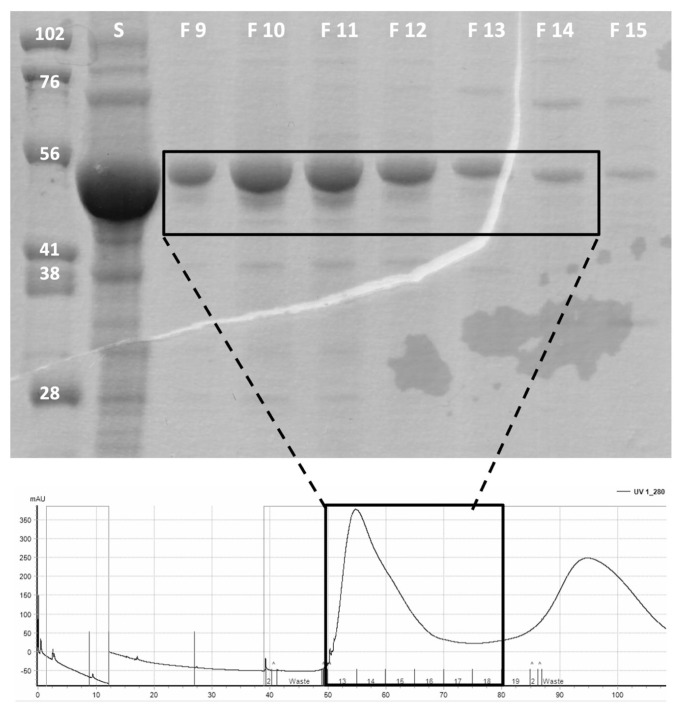
Size exclusion chromatography (SEC) purification of HER2-TKD and analysis of collected fractions by 12% SDS-PAGE. HER2-TKD protein was purified using SEC. In the chromatogram, fractions (F) 9–13 and 14–21 were collected as indicated by the box. These fractions were analyzed by 12% SDS-PAGE. A clear band corresponding to HER2-TKD (approximately 48 kDa) was predominantly observed in fractions 9–13, suggesting that the target protein was enriched in this range. The lane assignments are as follows: lane 1 contains the protein marker and lanes 2 to 6 correspond to fractions 9 to 13, respectively. A volume of 3 μL was loaded for all samples.

Soluble HER2-TKD was successfully overexpressed and the expression parameters were optimized for maximum yield. The best expression was obtained by induction with 0.4 mM IPTG at 37 °C and incubation for 7 h (Figure 1). Sarcosyl was used as a solubilizing agent to reduce inclusion body formation and a concentration of 1.5% [13] was applied (Figure 2). Despite the presence of a 6xHis tag in the construct, purification using Ni-NTA affinity chromatography was found to be inefficient, most likely due to poor binding or detergent interference. As a result, SEC was employed as an alternative purification strategy. Two peaks were observed in the SEC chromatogram, and SDS-PAGE analysis confirmed that HER2-TKD was present in the first peak (Figure 3). Fractions 9 to 13 from SEC were pooled and concentrated using 30-kDa Amicon filters.

### 3.2. Tag cleavage, affinity repurification, and concentration of HER2-TKD

SEC was employed as the primary purification method because Ni-NTA affinity purification was inefficient despite the presence of an N-terminal His_6_-SUMO tag. The SEC chromatogram contained two major peaks and SDS-PAGE analysis confirmed that HER2-TKD eluted mainly in fractions 9–13 (Figure 3). These fractions were pooled and processed for further steps.

**Figure 4:**
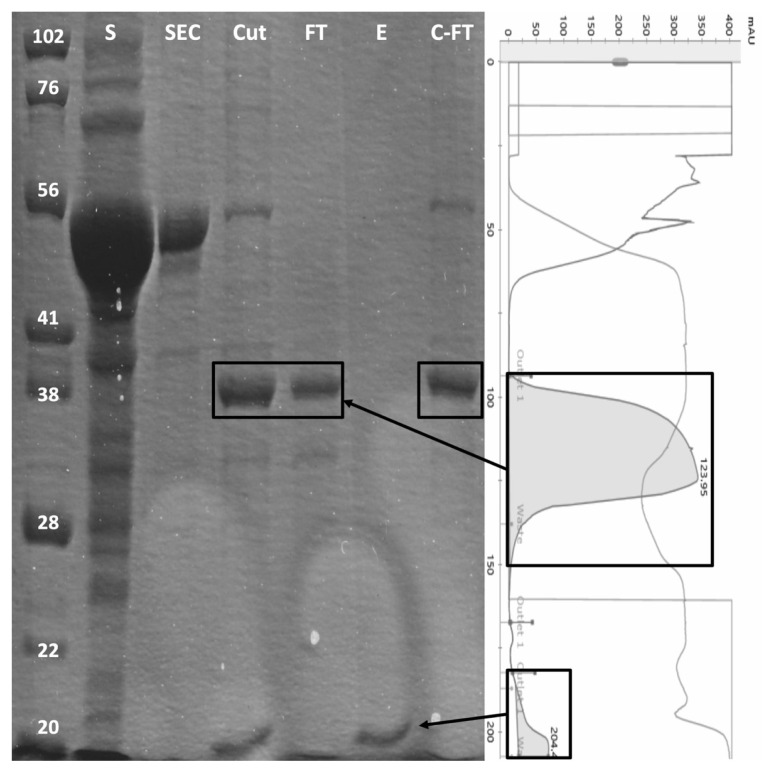
Purification of HER2-TKD via reverse Ni-NTA affinity after His_6_-SUMO tag cleavage. SDS-PAGE and chromatographic analysis of HER2 reverse protein after His_6_-SUMO tag cleavage and reverse Ni-NTA affinity purification; 12% SDS-PAGE gel was used to assess the purification of HER2-TKD (48 kDa) following cleavage of the His_6_-SUMO tag (35 kDa) by ULP protease. In the reverse Ni-NTA affinity setup, untagged HER2-TKD does not bind to the Ni-NTA resin and is found in the flow-through, whereas His_6_-tagged components—including uncleaved fusion protein, the cleaved SUMO tag, and the His-tagged protease—bind to the column and are later eluted. The gel includes the following samples: a molecular weight marker (lane 1), HER2-TKD supernatant (S) (lane 2), concentrated sample from size-exclusion chromatography (SEC) (lane 3), postcleavage sample containing tags (cut) (lane 4), flow-through (FT) fraction containing untagged HER2-TKD (lane 5), elution (E) fraction containing His-tagged components (lane 6), and a concentrated flow-through (C-FT) sample enriched in untagged HER2-TKD (lane 7). The chromatogram shown below the gel image depicts the purification profile, including UV absorbance at 280 nm, conductivity, and pH. The cleavage and purification were carried out at 4 °C for 40 min using a 5:1000 ULP:protein ratio.

Subsequent reverse Ni-NTA purification successfully separated the cleaved HIS-SUMO tag and ULP1 protease from untagged HER2-TKD, which was collected in the flow-through (Figure 4). Although cleavage was efficient, the solubility of HER2-TKD decreased after tag removal, limiting protein concentration to approximately 2.5 mg/mL. This reduction in solubility posed a challenge for downstream crystallization experiments, which typically require higher concentrations.

### 3.3. Crystal observation

**Figure 5:**
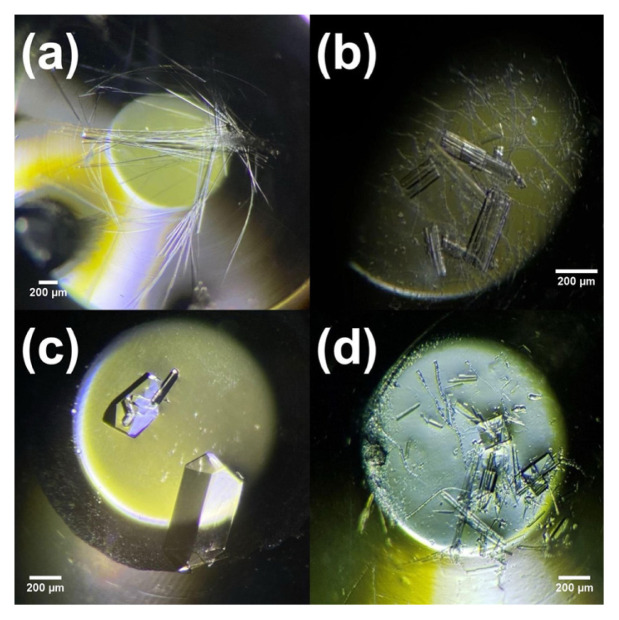
The HER2-TKD crystals obtained through cocrystallization with novel inhibitors. Microscope images of HER2-TKD protein crystals formed after SUMO tag removal and protein concentration. Cocrystallization was carried out in the presence of novel small-molecule inhibitors. (a) Needle-like crystals formed in the NRLBD-I #33 condition. (b) Crystals obtained in the Pact Premier II #22 condition. (c) Block-shaped crystals formed in the Wizard Synergy-II #1 condition. (d) Crystals grown in Crystal Screen I #24 condition.

Crystallization trials were performed with both SUMO-tagged and tag-removed HER2-TKD under approximately 3000 screening conditions at 4 °C and room temperature. Crystalline material generally appeared within 2–4 weeks. While several conditions produced crystalline structures, most droplets yielded salt crystals or amorphous precipitates. Needle-like or microblock crystals were observed in a small subset of conditions (Figure 5), but none diffracted to a resolution sufficient for structure determination.

Cocrystallization experiments with inhibitors (lapatinib, erlotinib, and compound B2) led to a slight increase in microcrystal formation, but diffraction-quality crystals were still not obtained. These outcomes highlight the intrinsic difficulty of crystallizing HER2-TKD and suggest that further optimization, such as screening additive libraries, stabilizing ligands, or alternative crystallization techniques, will be necessary to improve crystal quality.

## 4. Discussion

The present study represents an important step toward addressing the long-standing challenge of obtaining soluble HER2-TKD from bacterial expression systems. By using sarcosyl to eliminate inclusion bodies, we were able to recover HER2-TKD in a soluble form and establish a practical workflow that begins with inclusion body formation and proceeds through purification of the soluble protein. Although this strategy substantially improved solubility, it also revealed a notable limitation: Ni-NTA affinity purification remained inefficient, most likely because the detergent interfered with His-tag binding. Similar detergent-related disruptions in metal-affinity purification have been reported in other recombinant kinase studies [34–37], and our observations highlight the need for systematic evaluation of detergent-compatible purification strategies for HER family kinases.

Despite the improvement in solubility, crystallization of HER2-TKD remained difficult. More than 3000 crystallization conditions were screened, yet most droplets produced salt crystals or amorphous precipitates rather than protein crystals. This mirrors the complexity often described for receptor tyrosine kinase crystallization [16], including reports specifically noting the challenges associated with HER2-TKD [38]. Several factors likely contribute to this difficulty, such as the intrinsic conformational flexibility of the kinase domain, which complicates the formation of ordered crystal lattices [38,39].

Consistent with earlier structural studies, most high-resolution HER2-TKD structures available today were obtained in complex with stabilizing inhibitors such as lapatinib or neratinib (e.g., PDB IDs 3PP0, 3RCD) [38,40], whereas apo-form crystals seldom diffract to usable resolution. Many of these successful efforts relied on mammalian or insect cell systems to enhance folding and generate appropriate posttranslational modifications [38,40]. However, these systems are costly and less suitable for large-scale screening. In contrast, the goal of our work was to establish a cost-effective bacterial expression platform. Through optimized sarcosyl-based solubilization and downstream purification, we demonstrate that *E. coli* can serve as a viable and economical host for producing HER2-TKD for crystallization trials and drug-screening applications.

Overall, our findings offer practical insight into the production and purification of receptor tyrosine kinases, particularly in the context of recovering functional kinase domains from inclusion bodies. By expressing HER2-TKD in *E. coli* and implementing a sarcosyl-based solubilization strategy, we created a scalable and accessible system that can be adapted for structural and biochemical studies of HER2 and other ErbB family members.

**Figure 6:**
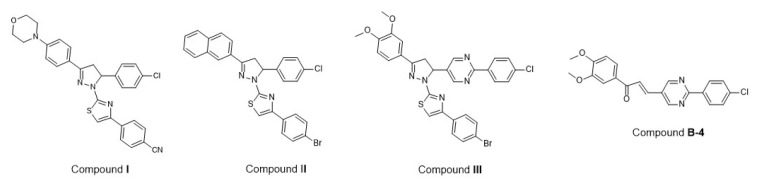
The chemical structures of compounds I, II, III, and B-4.

In parallel with these methodological advances, our research group has focused extensively on the rational design of small-molecule inhibitors targeting EGFR. Several pyrazoline–thiazole hybrid compounds developed in our laboratory (Figure 6) have shown promising anticancer activity and strong EGFR inhibition [11–13]. For example, compound I demonstrated superior NSCLC activity, compound II outperformed lapatinib, and B-4 exhibited notable potency within the same scaffold series [11–13]. Although these inhibitors were designed for EGFR, they illustrate a broader concept: small-molecule scaffolds can be rationally tuned to target different receptor tyrosine kinases within the ErbB family. The present HER2-TKD platform extends this concept by providing the protein infrastructure necessary for pursuing similar structure-based design efforts for HER2. By combining our prior experience in EGFR inhibitor development with the newly established HER2-TKD pipeline, we create a translational bridge supporting the future discovery of HER2-selective drug candidates.

The continued development of HER2-specific inhibitors highlights the clinical relevance of HER2-TKD in cancers such as breast cancer and NSCLC [1,6,23,34]. The successful production, purification, and initial crystallization trials presented here lay essential groundwork for obtaining high-resolution HER2–inhibitor complex structures and accelerating structure-based drug discovery. Our results are in agreement with recent structural and clinical reports emphasizing the significance of HER2 alterations in solid tumors [23,41]. Furthermore, this platform complements ongoing efforts to refine HER2 evaluation and guide patient stratification for HER2-targeted therapies [23,27,41].

Most importantly, the bacterial expression and purification platform established here supports downstream structure-based drug screening. Producing HER2-TKD in sufficient yield and purity enables systematic cocrystallization trials with small-molecule libraries, allowing determination of binding modes and experimental validation of computational docking predictions [42]. Integrating crystallography with in silico approaches creates a fast, cost-effective, and scalable pipeline that connects protein production directly to rational drug-design efforts. In this way, the HER2-TKD platform we report has the potential to accelerate the development of next-generation HER2-targeted therapeutics.

## Figures and Tables

**Table:** Condition List A. List of the crystallization screening conditions used in the study.

Natrix I	Wiz Synergy IV
Natrix II	Pact Premier I
Wizard I	Pact Premier II
Wizard II	PEG Ion I
Wizard II	PEG Ion II
Wizard IV	Salt RX I
Crystal Cryo I	Salt RX II
Crystal Cryo II	Proplex I
Crystal Screen I	Proplex II
Crystal Screen II	MembFAC
Crystal Screen II	Crystal Lite
Wizard Cryo I	PEG RX I
Wizard Cryo II	PEG RX II
Helix I	Multi XTAC
Helix II	Macro SOL
Midas I	3D Structure Screen
Midas II	Stura Footprint
NR-LBD I	Clear Strategy Screen I and II (pH 4.5)
NR-LBD II	Clear Strategy Screen I and II (pH 5.5)
PGA-LM I	Clear Strategy Screen I and II (pH 6.5)
PGA-LM II	Clear Strategy Screen I and II (pH 7.5)
Morpheus I	Clear Strategy Screen I and II (pH 8.5)
Morpheus II	Quick Screen / Ionic Liquid Screen
Structure I	GRID/NaCl – Na Malonate
Structure II	GRID/PEG 6000–Ammonium Sulfate
Index I	GRID/MPD – PEG LiCl
Index II	JENA BC Nuc Pro 1/2
Wiz Synergy I	JENA BC Nuc Pro 3/4
Wiz Synergy II	JCSG-I
Wiz Synergy III	JCSG-II
